# Iron Oxide Nanoparticle Uptake in Mouse Brachiocephalic Artery Atherosclerotic Plaque Quantified by T_2_-Mapping MRI

**DOI:** 10.3390/pharmaceutics13020279

**Published:** 2021-02-19

**Authors:** Rik P. M. Moonen, Bram F. Coolen, Judith C. Sluimer, Mat J. A. P. Daemen, Gustav J. Strijkers

**Affiliations:** 1 Department of Radiology and Nuclear Medicine, Maastricht University Medical Center, 6229 ER Maastricht, The Netherlands; rik.moonen@mumc.nl; 2 CARIM School for Cardiovascular Diseases, Maastricht University Medical Center, 6229 ER Maastricht, The Netherlands; judith.sluimer@maastrichtuniversity.nl; 3 Department of Biomedical Engineering and Physics, Amsterdam University Medical Centers, Amsterdam Cardiovascular Sciences, University of Amsterdam, 1105 AZ Amsterdam, The Netherlands; b.f.coolen@amsterdamumc.nl; 4 Department of Pathology, Maastricht University Medical Center, 6229 ER Maastricht, The Netherlands; 5 Department of Pathology, Amsterdam University Medical Centers, University of Amsterdam, Amsterdam Cardiovascular Sciences, 1105 AZ Amsterdam, The Netherlands; m.j.daemen@amsterdamumc.nl

**Keywords:** T_2_ mapping, quantitative MRI, USPIO, atherosclerosis, inflammation, macrophages

## Abstract

The purpose of our study was to monitor the iron oxide contrast agent uptake in mouse brachiocephalic artery (BCA) atherosclerotic plaques in vivo by quantitative T_2_-mapping magnetic resonance imaging (MRI). Female ApoE^−/−^ mice (*n* = 32) on a 15-week Western-type diet developed advanced plaques in the BCA and were injected with ultra-small superparamagnetic iron oxides (USPIOs). Quantitative in vivo MRI at 9.4 T was performed with a Malcolm-Levitt (MLEV) prepared T_2_-mapping sequence to monitor the nanoparticle uptake in the atherosclerotic plaque. Ex vivo histology and particle electron paramagnetic resonance (pEPR) were used for validation. Longitudinal high-resolution in vivo T_2_-value maps were acquired with consistent quality. Average T_2_ values in the plaque decreased from a baseline value of 34.5 ± 0.6 ms to 24.0 ± 0.4 ms one day after injection and partially recovered to an average T_2_ of 27 ± 0.5 ms after two days. T_2_ values were inversely related to iron levels in the plaque as determined by ex vivo particle electron paramagnetic resonance (pEPR). We concluded that MRI T_2_ mapping facilitates a robust quantitative readout for USPIO uptake in atherosclerotic plaques in arteries near the mouse heart.

## 1. Introduction

Atherosclerosis is a disease that progresses silently over many years. A first sign of its presence often is a potentially lethal event such as a myocardial infarction or stroke, caused by rupture of an atherosclerotic plaque and exposure of thrombogenic material to the bloodstream. Risk of plaque rupture, however, remains difficult to predict. It is likely a multifactorial combination of different plaque features that contribute to the overall risk of rupture, including the thickness of the fibrous cap and the presence of a large lipid core, as well as hemodynamic features, such as a high blood pressure and high wall shear stress [1,2,3,4,5]. Plaque inflammation is considered one of the hallmarks of plaque rupture risk, as inflammatory cells excrete enzymes that break down the extracellular matrix, leading to plaque destabilization [6,7]. Tools for in vivo imaging of plaque inflammation are therefore highly desired, for diagnostic purposes as well as for monitoring responses to medication.

For this reason, MRI inflammation imaging facilitated by nanoparticulate iron oxide contrast agent has been extensively studied. Intravenously injected ultra-small superparamagnetic iron oxide particles (USPIOs) have been shown to accumulate in macrophage-rich plaques in rabbits [8,9] and humans [10,11,12]. The same technique was successfully applied in ApoE^−/−^ mice to noninvasively monitor age-related plaque progression and anti-inflammatory drug treatments [13,14,15]. Clinical potential has been demonstrated by showing the feasibility of long-term follow-up studies [16] and treatment monitoring [17,18]. In another study, an association between plaque inflammation detected by USPIO MRI and subsequent clinical events was observed. However, the study design lacked the statistical power to prove this predictive value [19].

The technique of USPIO-enhanced MRI of plaque inflammation would highly benefit from standardized quantitative readout methods. Standardization will allow for better comparison of results among different research centers and MRI systems and ultimately a better comparison of therapeutic procedures aimed at reducing atherosclerotic plaque inflammation. Quantitative imaging, in principle, allows for quantification of iron concentration. Quantitative imaging of USPIO in atherosclerosis mostly focused on techniques such as T_2_* mapping [13,14,17,20,21,22] and susceptibility gradient mapping [15,23]. However, in vasculature that is heavily affected by cardiac and respiratory motion, such as the arteries near the heart, the latter techniques are prone to movement artifacts, which negatively affects their quantitative power. Quantitative T_2_ mapping has the advantage that it can be introduced using a motion-insensitive T_2_-preparation module in combination with a standard high-resolution imaging sequence, which makes T_2_ mapping potentially more suitable for quantification of atherosclerosis in arteries near the heart [24,25,26].

The aim of the current study was therefore to monitor uptake of iron oxide contrast agent in mouse brachiocephalic artery (BCA) atherosclerotic plaque in ApoE^−/−^ mice in vivo by quantitative T_2_-MRI. Quantitative imaging in the heart region is demanding because of a combination of cardiac and respiratory motion, and therefore robust acquisition strategies are required. In future studies, this method can be applied to investigate, for example, therapy response. The ApoE^−/−^ is the most used atherosclerotic mouse model, and it develops atherosclerotic plaques throughout the major arteries, a process that is accelerated by a Western-type diet [27]. The brachiocephalic artery (BCA) plaque, which is one of the first advanced atherosclerotic plaques to form in this model [28], was selected because of its close proximity to the heart. The novelty lies in the use of a segmented MLEV-prepared T_2_-mapping sequence that was previously developed for use in mouse cardiac imaging and proved very robust to quantify T_2_ of myocardial tissue [29]. For the first time, this T_2_-mapping method was used for repeated quantitative imaging of BCA plaques in mice before and after USPIO injection. The experiments were validated against iron concentration determined by ex vivo particle electron paramagnetic resonance (pEPR).

## 2. Materials and Methods

### 2.1. Animal Experiments

All procedures regarding animals were approved by the ethical review committee of Maastricht University and were performed according to Dutch national law and the guidelines set by the institutional animal care committee, accredited by the national department of health. Female C57bl/6 ApoE^−/−^ mice (Charles River Laboratories, Maastricht, The Netherlands) were placed on a Western-type diet with 0.25% cholesterol (4012.6 Purified diet W, ABdiets) ad libitum at 7 weeks of age. The first MRI session was performed 15 weeks after the start of the diet.

The mice were anesthetized with isoflurane (1–2%) in medical air (0.4 L/min), and a catheter was inserted into the tail vein for contrast agent injection. Subsequently, they were placed in a prone position in a cradle equipped with an anesthesia mask and fixed gold-plated electrocardiogram (ECG) electrodes connected to an ECG triggering system (Small Animal Instruments, Stony Brook, NY, USA). ECG paste was applied to the front paws, which were taped to the electrodes. Body temperature was monitored with a rectal probe and maintained at approximately 37 °C with a warm-water pad. Respiratory rate was kept at 50–60 bpm and monitored with a pressure balloon. At the end of the first MRI session, a 150 µL bolus containing 1.0 mmol/kg Sinerem® (ferumoxtran, Guerbet, France) was injected via the catheter. Ferumoxtran is composed of iron oxide particles of about 4–6 nm, covered with dextran, resulting in a hydrodynamic diameter of about 20–40 nm. MRI sessions were repeated at day 1 (*n* = 5) or at both days 1 and 2 (*n* = 27) after injection.

Directly after the last MRI session, a subcutaneous injection of buprenorphine hydrochloride (0.1 mg/kg, Sigma-Aldrich, Zwijndrecht, The Netherlands) for analgesia was administered. After 15 min, the mice were euthanized by incision of the diaphragm and the vena cava was transected. Tissue perfusion was performed with 5 mL 0.9% NaCl followed by 5 mL 1% paraformaldehyde (Sigma-Aldrich, Zwijndrecht, The Netherlands) injected directly into the left ventricle. Subsequently, the heart with aortic arch and carotid and subclavian arteries were excised, pinned onto a piece of cork and fixed for 24 h in 1% paraformaldehyde before storage in 70% ethanol.

### 2.2. MRI Protocol

**Figure 1:**
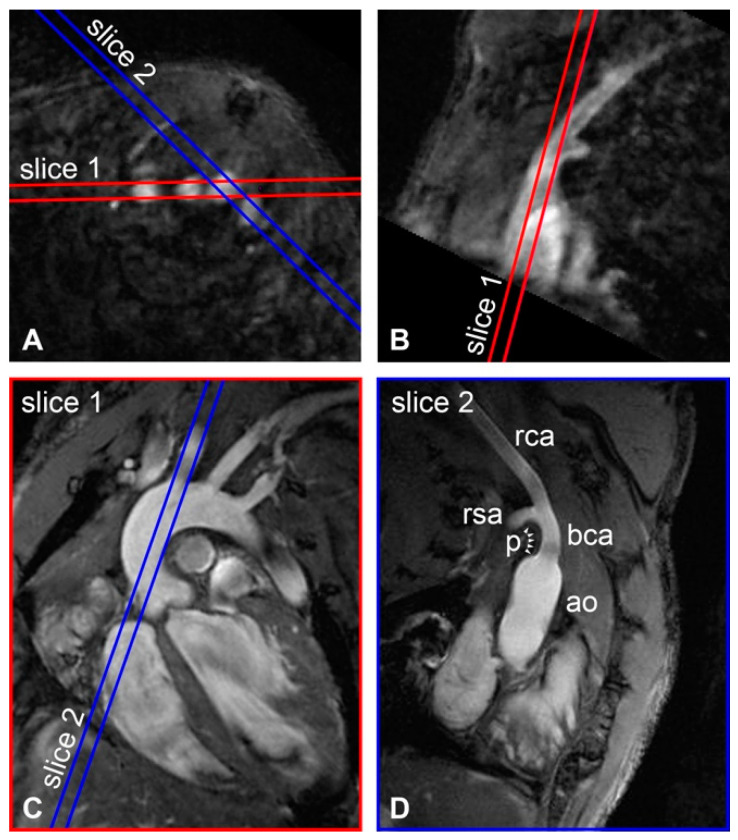
Slice planning. (**A**) Axial oblique and (**B**) sagittal oblique slices reconstructed from 3D-TOF-FLASH were used for planning T_1_-weighted anatomical reference images with (**C**) aortic arch view and (**D**) brachiocephalic artery (BCA) view. ao = ascending aorta; bca = BCA; rca = right carotid artery; rsa = right subclavian artery; *p* = atherosclerotic plaque (arrowheads).

Measurements were performed with a 9.4 T animal scanner equipped with a 72-mm-diameter volume transmit coil and a four-element mouse cardiac phased-array surface receiver coil (Bruker BioSpin, BioSpec, Ettlingen, Germany). For planning purposes, a 3D fast low-angle shot time-of-flight was acquired of the chest region, with the following parameters: sequence = 3D-FLASH-TOF, echo time (TE) = 2.5 ms, repetition time (TR) = 17 ms, field of view (FOV) = 20 × 20 × 10 mm^3^, matrix = 200 × 200 × 100, number of averages (NA) = 2, flip angle (FA) = 20°, and acquisition time = 11 min 30 s. Axial and longitudinal cross-sectional slices of the BCA were reconstructed from the 3D dataset (Figure 1A,B). These were used for planning of a slice containing the aortic arch with its bifurcations (Figure 1C), which was subsequently used for planning of the brachiocephalic artery view (Figure 1D). The BCA view is a longitudinal slice of the ascending aorta, right carotid artery and right subclavian artery, resulting in a slice through the thick center part of the advanced plaque that was present in the inner curvature of the brachiocephalic-/subclavian-artery bifurcation of these mice (Figure 1D). Both the aortic arch view and BCA view images were acquired with a respiratory-gated, ECG-triggered T_1_-weighted FLASH sequence, with: TE = 3.2 ms, TR = 1 R-R interval, FOV = 20 × 20 mm^2^, matrix = 400 × 400, slice thickness = 0.5 mm, NA = 6, FA = 40°, and acquisition time = 5 min. Additionally, for three mice, two sets of black-blood images were acquired axial to the BCA. These sets respectively contained two and three slices that were interleaved to span the full length of the plaque and served to validate the plaque region of interest (ROI) drawn on the BCA view. Black-blood imaging was achieved with inversion recovery (IR)-FLASH with the same settings as for the BCA view FLASH protocol and a mean inversion time of 69.6 ± 6.3 ms, adjusted to the heart rate of the individual mice.

T_2_ mapping of the BCA view was performed with a T_2_-prepared segmented gradient echo sequence, as previously published [29]. In short, the T_2_ preparation consisted of excitation with a composite 90° pulse followed by a series of composite 180° refocusing pulses with alternating phase (+ or −) according to an MLEV scheme. This specific pulse train diminishes sensitivity to both B_0_ and B_1_ inhomogeneities. Multiple TEs were obtained by varying the number of refocusing pulses in sets of four, following an MLEV16 scheme. Resulting effective echo times were TE_eff_ = 0.9, 8.8, 14.7, 21.3, 28.5, and 34.9 ms. Since pulse durations and echo spacing were set as short as considered possible given the hardware restrictions, minor deviations from these TE_eff_ values were caused by differences in pulse calibration, which are inherent to variable coil loading. These deviations were accounted for during data analysis.

The brachiocephalic artery is located close to the heart (~2.5 mm), and imaging was challenging due to cardiac and respiratory motion, as well as high blood-flow rates. Therefore, the T_2_-mapping sequence was respiratory-gated and ECG-triggered with the acquisition during the end-diastolic phase of the cardiac cycle. Acquisition was maintained at the same point in the cardiac cycle, regardless of the duration of the preceding T_2_ preparation. Therefore, the T_2_ preparation was performed in a global fashion to avoid adverse effects of motion. In order to confine the acquisition to the end-diastolic time window, the acquisition was split into 40 segments of 5 echoes each. The other parameters of the gradient echo readout were: TE = 2.1 ms, TR = 4.1 ms, segment TR = 2000 ms, FOV = 20 × 20 mm^2^, acquisition matrix = 200 × 200, reconstructed matrix = 400 × 400, NA = 4, FA = 30°, and total acquisition time = 32 min.

### 2.3. MRI Data Analysis

Image analysis was performed using a custom-built algorithm in Mathematica 8.0 (Wolfram Research, 2010, Champaign, IL, USA). T_2_ maps were generated by pixel-wise fitting of the signal intensity as function of TE_eff_ with a mono-exponential decay function. An ROI was manually drawn around the plaque on the image with shortest TE_eff_ for each of the three time points. To validate this plaque ROI drawn on the BCA view, ROIs encompassing the plaque were also drawn on the axial black-blood images. From the relative 3D orientation of these slices, the intersections of axial ROIs with the BCA view were determined, and agreement with these perpendicular ROIs was inspected visually. A filter was applied to select all pixels in the plaque ROI with an R^2^ of fit larger than 0.7. Subsequently, the mean plaque T_2_ was determined by averaging of R_2_ = 1/T_2_ over all remaining pixels. Additionally, ΔR_2_, the difference between pre- and post-injection R_2_, was calculated because this parameter is directly proportional to the difference in iron oxide concentration. The plaque area on MRI was determined by multiplying the total pixel count in the plaque ROI with the in-plane pixel area (0.05 × 0.05 = 0.0025 mm^2^).

### 2.4. Histology and Immunohistochemistry

Histological analysis was performed on aortas of 21 mice sacrificed at day 2 after injection. After paraffin embedding, 40–80 consecutive longitudinal sections of 4 µm thick were cut to cover the full width of the brachiocephalic artery. After overnight drying at 56 °C, every 5th section was stained with hematoxylin and eosin (HE), for nuclei and cytoplasm, respectively. Four consecutive HE-stained sections in the center of each plaque were selected to determine the mean histology plaque area. Adjacent sections from the center region of the plaque were stained for macrophages with MAC3. After heat-induced antigen retrieval (Target Retrieval, DAKO Agilent, Amstelveen, The Netherlands) and blocking with 10% rabbit serum, the sections were incubated overnight at room temperature with rat anti-MAC3 (Becton Dickenson, Vianen, The Netherlands). They were then subsequently incubated with rabbit anti-rat-biotin (DAKO Agilent, Amstelveen, The Netherlands) and alkaline phosphatase coupled avidin-biotin-complex reagent (Vector/Brunschwig Chemie, Amsterdam, The Netherlands), developed with vector red substrate and counterstained with hematoxylin. Sections were imaged with bright-field microscopy at 10× magnification. All histological image processing was performed using a custom-built algorithm in Mathematica 8.0.

### 2.5. pEPR

The iron content in the aortic arch of two other groups of mice euthanized at day 1 (*n* = 5) and day 2 (*n* = 6) after injection was assessed with pEPR. Aortas of mice that received the same diet but were not injected with Sinerem served as control (*n* = 3). The fixed aortic arches were dissected in the same way as those taken for histology, and were placed in 250 µL vials with 70% ethanol. Measurements were performed with a Pepric Particle Spectrometer 3-V2 (Pepric NV, Leuven, Belgium). During the measurements, a radiofrequency (RF) field of 300 MHz and a magnetic field of 10 mT were employed. The pEPR measurement voltage was directly proportional to the particles in resonance with the RF-field [30]. Hence, there was no contamination from iron in tissue, blood, and intraplaque hemorrhages, as would be the case for a more conventional inductively coupled plasma mass spectrometry (ICP-MS) determination of iron content. A calibration with a series of samples with a known concentration of Sinerem was performed to obtain the conversion factor between voltage and iron quantity.

### 2.6. Statistics

Statistical analysis was performed with the Statistical Package for the Social Sciences (SPSS) software version 22.0 (IBM Corp., 2013, Armonk, NY, USA). The Shapiro–Wilk test was applied to test the normality of each group. Correlations were tested using Pearson’s correlation coefficient. Linear regression through the origin was applied to determine the relation between the plaque areas on MRI and histology. Two cases were excluded from area analysis; one because of folded histological slices and one because of an incorrect slicing angle. T_2_ values for different pre- and post-injection time points were compared with one-way ANOVA for repeated measures and post hoc Bonferroni. ΔR_2_ values were compared with a two-sided paired-samples t-test. The amount of Fe as determined by pEPR was compared between groups with two-sided independent samples t-tests. The differences in pEPR Fe and ΔR2 between time points were tested with multivariate ANOVA. Unless stated otherwise, all values are reported as mean ± standard error (SE); in case of repeated measures, SE was corrected for between-subject variations. The significance level for all statistical tests was set at *α* = 0.05.

## 3. Results

**Figure 2:**
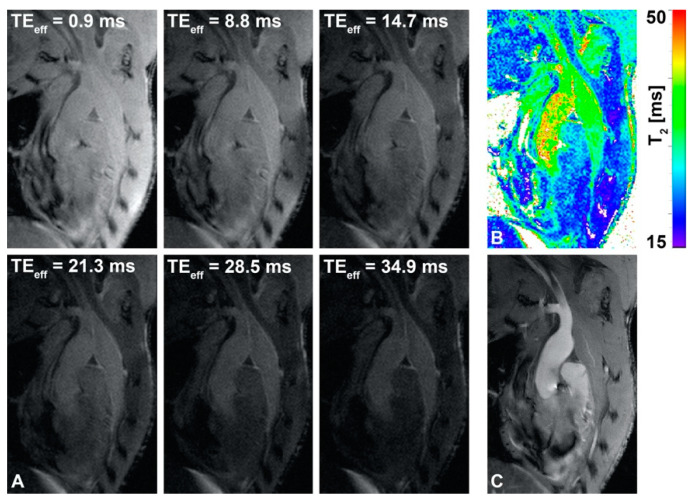
T_2_-weighted images and T_2_-value map. (**A**) T_2_-weighted images with 6 different effective echo times (TE_eff_) and (**B**) false-color T_2_-value map of BCA view pre-injection. (**C**) T_1_-weighted anatomical reference image.

**Figure 3:**
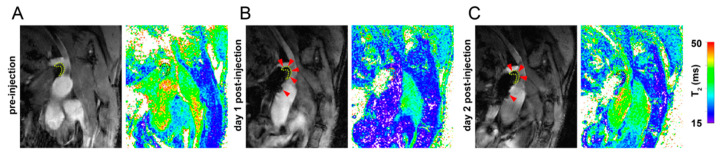
T_1_-weighted anatomical reference images and T_2_-value maps from the same animal (**A**) before and at (**B**) day 1 and (**C**) day 2 after injection of Sinerem. Arrowheads point to locations of contrast agent uptake in the plaques.

The protocol that was developed used a convenient planning scheme to attain a reproducible slice orientation (Figure 1). Consequently, high-resolution T_1_-weighted anatomical images with high quality could be routinely acquired (Figure 1D). Figure 2A shows T_2_-weighted BCA view images of a mouse before Sinerem injection at six different TE_eff_. No motion artifacts were observed and anatomical registration of images with different TE_eff_ was excellent, allowing pixel-wise fitting to generate a T_2_-value map (Figure 2B). T_1_-weighted images, such as those presented in Figure 2C, offer a higher blood–plaque and blood–tissue contrast, and were used as the anatomical reference. Examples of T_1_-weighted anatomical reference images and corresponding T_2_-value maps at different pre- and post-injection time points are presented in Figure 3. The slice planning proved reproducible, and this facilitated monitoring of T_2_ values in the plaque over a prolonged period of time. In the post-injection T_1_-weighted images, the iron oxide contrast agent appears as dark spots, which can be observed inside the plaque as well as in the ascending aorta artery wall (Figure 3).

**Figure 4:**
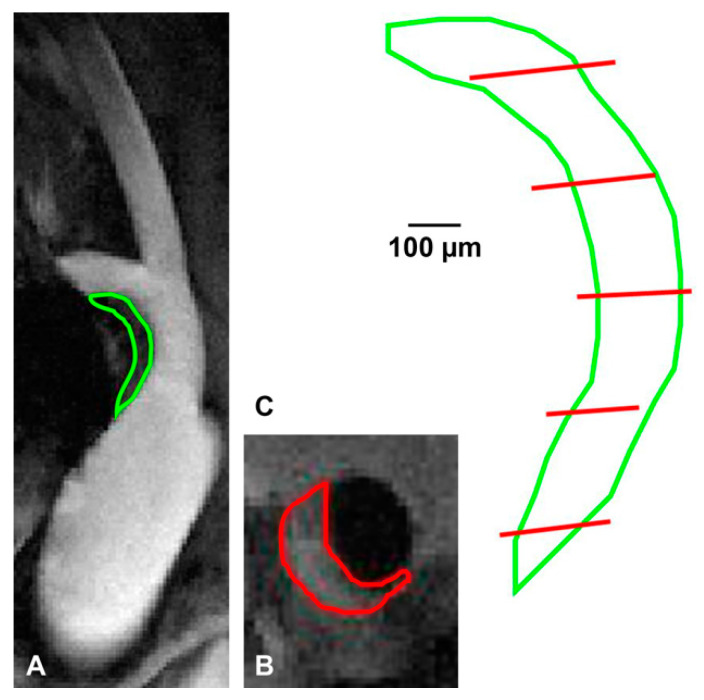
Validation of plaque ROI. (**A**) Example of plaque ROI drawn on BCA view as used for analysis. (**B**) Example of plaque ROI on axial view black-blood image of the same mouse. (**C**) Intersections of axial ROIs with the BCA view image plane (red) superimposed on the longitudinal plaque ROI (green).

In order to determine the mean plaque T_2_, an ROI was drawn around the plaque, as shown on a T_1_-weighted reference image in Figure 4A. These ROIs were validated in three mice. Plaque ROIs were drawn on five T_1_-weighted black-blood images, oriented axial to the BCA (Figure 4B). Using the 3D position and orientation of the imaging planes, the intersections of the axial ROIs with the BCA view could be determined and were superimposed onto the longitudinal ROI (Figure 4C). These ROIs showed good correspondence as assessed by visual inspection.

**Figure 5:**
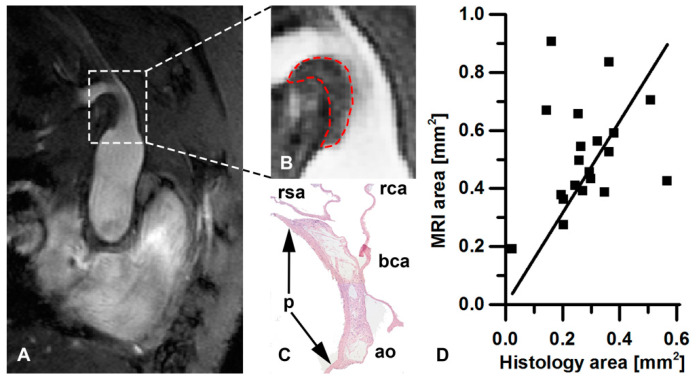
Comparison of MRI and histology. (**A**) T_1_-weighted anatomical reference image of BCA view and (**B**) enlarged view with enhanced contrast of plaque region with ROI (dashed red line). (**C**) Plaque region of the same mouse as observed on HE-stained histology. ao = aortic arch; bca = BCA; rca = right carotid artery; rsa = right subclavian artery; *p* = atherosclerotic plaque (extends between arrows). (**D**) Scatterplot of plaque area on MRI versus plaque area on HE-stained histology. The solid line represents the linear fit of the data (*R*^2^ = 0.87).

**Figure 6:**
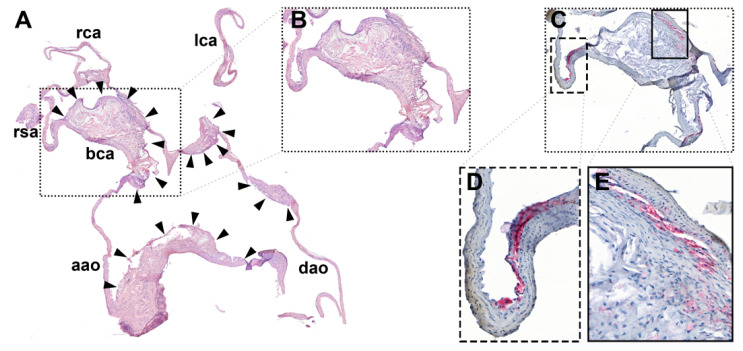
(**A**) Representative HE-stained slice of the aortic arch with the BCA and the left carotid artery. Magnifications of the BCA plaque region (boxes) with (**B**) HE and (**C**–**E**) MAC3 stains performed on adjacent slices. HE: pink = cytoplasm; blue = nuclei; MAC3: red = macrophages; blue = nuclei and cytoplasm; aao = ascending aortic; dao = descending aorta; bca = BCA; lca/rca = left/right carotid artery; lsa/rsa = left/right subclavian artery; solid arrowheads = BCA plaque; open arrowheads = other plaques.

Figure 5 shows a comparison of the BCA plaque observed by MRI and histology. In both the T_1_-weighted image of the plaque (Figure 5B) and the HE-stained sections (Figure 5C), the plaque extends from the caudal end of the BCA along its entire length into the left subclavian artery. The scatterplot presented in Figure 5D shows the correlation of ROI area to the plaque area as determined from histology (*r* = 0.446, *p* = 0.028). A linear fit through the origin yielded a slope of 1.47 (*R*^2^ = 0.87). The smaller area on histology was probably due to the well-known shrinkage of the tissue during fixation. An example of two consecutive sections with HE and MAC3 macrophage staining, respectively, is shown in Figure 6.

**Figure 7:**
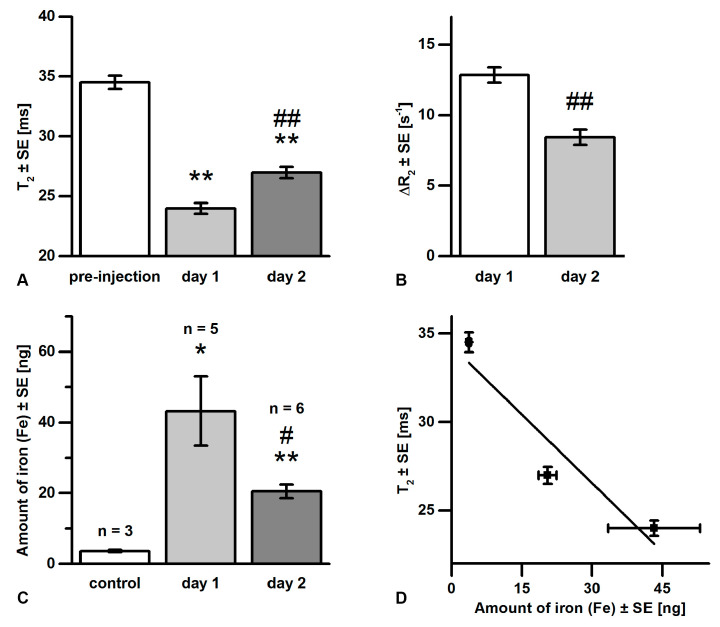
Bar graphs representing mean values ± standard error (SE). (**A**) Mean T_2_ values of plaque ROI at three time points (*n* = 27). (**B**) Average ΔR_2_ values of plaque ROI at day 1 and 2 (*n* = 27). (**C**) Mean amount of iron (Fe) in the aortic arch determined by pEPR for three groups sacrificed at different time points. Significant differences with pre-injection or control values are marked with * (*p* < 0.05) or ** (*p* < 0.001). Significant differences with day 1 values are marked with # (*p* < 0.05) or ## (*p* < 0.001). (**D**) Mean R_2_ values versus iron content. The solid line represents a linear fit (*R*^2^ = 0.86).

Mean T_2_ and ΔR_2_ values averaged over the plaque ROI are presented in Figure 7A,B, respectively. T_2_ values significantly decreased from a pre-injection value of 34.5 ± 0.6 ms to 24.0 ± 0.4 ms after 1 day, and partially recovered to an average T_2_ value of 27.0 ± 0.5 ms at 2 days post-injection (*n* = 27). T_2_ values at the two time points after injection correlated with each other (*r* = 0.464, *p* = 0.022), indicating that both were related to the total amount of iron oxide uptake. As a quantitative parameter directly proportional to the amount of iron oxide, ΔR_2_ was determined to be 12.9 ± 0.5 s^−1^ on day 1 and decreased to 8.4 ± 0.5 s^−1^ on day 2. ΔR_2_ values at both days were strongly correlated to the T_2_ values at the corresponding time point (*r* = −0.778 and *r* = −0.772, respectively, *p* < 0.001) and to each other (*r* = 0.522, *p* = 0.009), but not to the pre-injection T_2_ value (*r* = 0.388, *p* = 0.061 and *r* = 0.371, *p* = 0.074, respectively). This confirmed that the increase of R_2_ was independent of the native plaque T_2_, and the effect could be attributed to the uptake of iron oxide.

The bar graph in Figure 7C displays the amount of iron detected ex vivo in the aortic arch by pEPR. The control group (*n* = 3) yielded a level of 3.5 ± 1.6 ng, which was at the lower limits of detection. The aortas of the group sacrificed one day after injection (*n* = 5) had an Fe content of 43.2 ± 9.8 ng, which was significantly higher than the 20.5 ± 1.9 ng measured for the group at day 2 (*n* = 6). The correlation of Fe content with ΔR_2_ values of individual aortas, as tested by multivariate ANOVA of the combined post-injection groups (*n* = 11), was non-significant (*p* = 0.098). However, group-averaged Fe content was clearly linearly (*R*^2^ = 0.86) related to R_2_, as shown in Figure 7D.

## 4. Discussion

USPIOs are taken up by macrophages residing in the plaque or in blood monocytes that infiltrate the atherosclerotic plaque [31]. In this study, we showed that quantitative T_2_-MRI using an MLEV-prepared T_2_-mapping sequence allowed in vivo monitoring of iron oxide nanoparticulate contrast agent in mouse atherosclerotic plaques. We specifically demonstrated consistent and robust quantitative T_2_-value imaging of the brachiocephalic artery plaque before and after USPIO uptake. One day after injection of USPIO, a significant decrease in plaque T_2_ as compared to baseline was observed, which partially recovered after 2 days.

The MLEV-prepared sequence was successfully applied for high-resolution imaging of the BCA plaque. The triggered approach allowed excellent anatomical registration of images acquired during a single scan session, and the robust slice-planning procedure ensured consistency over multiple sessions. The accuracy of in vivo plaque delineation was confirmed by the strong correlation of plaque area on MRI and histology.

Plaque iron content was measured using pEPR and tested for correlation with R_2_ and ΔR_2_ values. In pEPR measurements, all iron oxide nanoparticles in the sample contribute to the signal, thereby providing a good validation for the MRI data. However, pEPR was performed on the whole sample containing the aortic arch and some of its bifurcations, and therefore was less specific for the BCA plaque. Nevertheless, a clear relation between iron content and changes in BCA plaque R_2_ values was observed (Figure 7).

It remains to be tested whether USPIO uptake quantified by T_2_ mapping correlates with plaque progression and treatment response [13,14,15,17]. As a possible next step, post-injection T_2_ and ΔR_2_ values could therefore be related to plaque inflammation and macrophages in studies incorporating age-related progression or therapeutic effects. For these studies, there are several important factors to keep in mind when interpreting T_2_ changes as a measure for USPIO uptake and plaque inflammation. For one, R_2_ = T_2_^−1^ is linearly related to the concentration of superparamagnetic particles when dispersed in a solution; however, upon uptake into cells, the particles may become clustered, and their relaxivity could change [32,33,34]. This would make straightforward quantification of local iron concentration in tissue difficult. Furthermore, although the USPIO are predominantly macrophage-associated, local iron concentration is not a direct measure of macrophage number. Yet, it is the result of the cumulative effect of macrophage content, subtype, and activity, as well as plaque permeability and contrast-agent pharmacokinetics.

The sequence design with a global T_2_-preparation module and signal acquisition during end-diastole is suitable for translation to patients and can be expected to enable similarly stable quantitative imaging in the highly dynamic region around the human heart [35,36]. The longer end-diastolic phase of humans is beneficial for translation and might be used to compensate for the lower inherent resolution of clinical scanners. On the other hand, SAR limitations might prevent the use of many repeated composite refocusing pulses during T_2_ preparation; however, local B_0_ and B_1_ inhomogeneities will be less at clinical field strengths. Another important aspect to be considered during clinical translation is the contrast agent. Sinerem is no longer available, as its production has been discontinued by the manufacturer. A promising USPIO of similar size is ferumoxytol, an FDA-approved supplement for treatment of iron deficiency that can also be used as an MRI contrast agent [37,38]. Preclinical studies showed that it induces larger signal changes in the plaque than Sinerem [39,40]. Ferumoxytol has been used to image myocardial inflammation [41,42], and is being evaluated for monitoring inflammation in carotid atherosclerosis [20,22,43].

## 5. Conclusions

In conclusion, the T_2_-mapping protocol presented in this study provided robust quantitative imaging of nanoparticulate iron oxide uptake in atherosclerotic plaques in blood arteries near the heart. Future studies should assess the sensitivity of this method for the monitoring of plaque progression and treatment response.

## Data Availability

The data presented in this study are available on request from the corresponding author.
